# Proteomic profiling of plasma biomarkers in acute ischemic stroke due to large vessel occlusion

**DOI:** 10.1186/s12967-019-1962-8

**Published:** 2019-07-01

**Authors:** Chuan Qin, Xin-Ling Zhao, Xiao-Tong Ma, Luo-Qi Zhou, Long-jun Wu, Ke Shang, Wei Wang, Dai-Shi Tian

**Affiliations:** 1 Department of Neurology, Tongji Hospital, Tongji Medical College, Huazhong University of Science and Technology, Wuhan, 430030 People’s Republic of China; 2 Department of Neurology, Mayo Clinic, Rochester, MN 55905 USA

**Keywords:** Ischemic stroke, Large vessel occlusion, Collateral circulation, Proteomics, Biomarkers

## Abstract

**Background:**

Acute ischemic stroke (AIS) due to large vessel occlusion (LVO) is a devastating cerebrovascular disorder, which could benefit from collateral circulation. Proteins associated with acute LVO pathogenesis and endothelial function may appear in blood samples of AIS patients due to LVO, thus permitting development of blood-based biomarkers for its diagnosis and prognosis.

**Methods:**

This study is a single-center, retrospective, observational case–control trial. Consecutive patients who presented at the Department of Neurology of Tongji Hospital were recruited from July 2016 to April 2018. In the discovery phase, a proteomic approach with iTRAQ-based LC–MS/MS was used to investigate the altered proteomic pattern in plasma from patients with AIS due to LVO. In the validation study, Western blots was used to identify biomarkers associated with stroke diagnosis as well as their prognostic value associated with different collateral statuses.

**Results:**

For this exploratory study, the proteomic analysis of plasma from 40 patients with AIS due to LVO and 20 healthy controls revealed seven differentially expressed proteins with a 1.2/0.83-fold or greater difference between groups. The four elevated proteins, PPBP (1.58 ± 0.78 vs 0.98 ± 0.37; P < 0.001), THBS1 (1.13 ± 0.88 vs 0.43 ± 0.26; P < 0.001), LYVE1 (1.61 ± 0.55 vs 0.97 ± 0.50; P < 0.001), and IGF2 (1.19 ± 0.42 vs 0.86 ± 0.24; P < 0.001), were verified by Western blots analysis in an independent cohort including 33 patients and 33 controls. A strong interaction was observed between the four-protein panel and the diagnosis of AIS due to LVO (AUC 0.947; P < 0.001). Furthermore, IGF2, LYVE1, and THBS1 were closely associated with collateral status (IGF2 0.115, 95% CI 0.016–0.841, P = 0.033; LYVE1 0.183, 95% CI 0.036–0.918, P = 0.039; THBS1 4.257, 95% CI 1.273–14.228, P = 0.019), and proved to be independent predictors of good outcome (IGF2 0.115, 95% CI 0.015–0.866, P = 0.036; LYVE1 0.028, 95% CI 0.002–0.334, P = 0.005; THBS1 3.294, 95% CI 1.158–9.372, P = 0.025) at a 3-month follow-up.

**Conclusions:**

The identified 4-biomarker panel could provide diagnostic aid to the existing imaging modalities for AIS due to LVO, and the prognostic value of IGF2, LYVE1, and THBS1 was proved in predicting functional outcomes related to collateral status.

*Trial registration* ClinicalTrials.gov NCT 03122002. Retrospectively registered April 20, 2017. URL of trial registry record: https://www.clinicaltrials.gov/ct2/show/NCT03122002?term=NCT+03122002&rank=1

**Electronic supplementary material:**

The online version of this article (10.1186/s12967-019-1962-8) contains supplementary material, which is available to authorized users.

## Background

Large-vessel occlusions (LVOs) cause more than one-third of acutely presenting ischemic stroke events, and are associated with severe functional deficits [1]. Collateral circulation in patients with AIS may sustain the hypoperfused tissue at risk [2]. Good collaterals have been associated with improved recanalization, smaller infarct volume, and better clinical outcome [2]. We hypothesized that for patients with AIS due to LVO, related biomarkers with different collateral status could be potent predictors of functional outcomes at follow-up.

A novel quantitative proteomic technology, isobaric tagging for relative and absolute quantitation (iTRAQ), has recently become a powerful tool to characterize differentially expressed proteins and identify the biomarkers for central nervous systems disorders, particularly for stroke [3–5]. The new technology allows broad protein biomarker screenings, offering the hope to better understand the pathophysiological mechanisms underlying AIS due to LVO. Proteins associated with acute LVO pathogenesis and endothelial function may appear in blood samples of AIS patients due to LVO, thus permitting development of blood-based biomarkers for its diagnosis and prognosis.

In this study, we sought to discover potential biomarkers associated with AIS due to LVO by using proteomic technology with iTRAQ-based LC–MS/MS. Western blots analysis was used to further validate those candidate proteins for diagnosis of AIS due to LVO, and to evaluate their prognostic value related to different collaterals. This could potentially identify a proteomic signature that aids in understanding the pathophysiological pathway underlying the link between acute large vessel occlusion, different collateral status, and functional outcomes at follow-up.

## Methods

### Study design and subjects

**Fig. 1:**
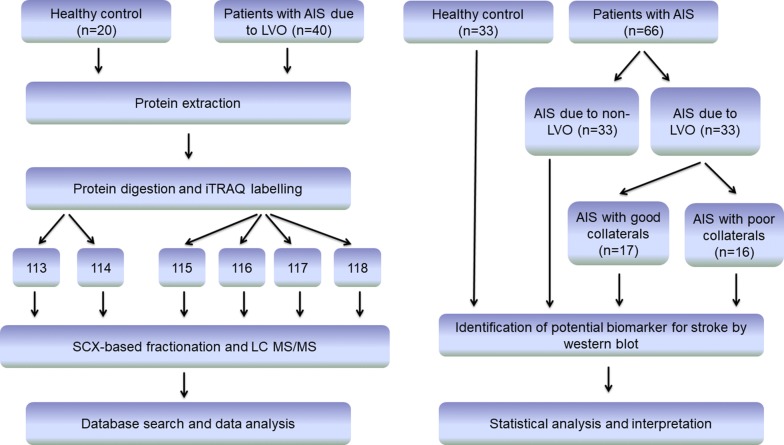
Flow diagram of the study design. Discovery phase: proteomic analysis of AIS patients due to LVO (4 groups, 10 individuals pooled in a group) versus healthy controls (2 groups, 10 individuals pooled in a group). Validation phase: Western blot analysis of selected plasma biomarkers in healthy controls (n = 33), patients with AIS due to non-LVO (n = 33), and patients with AIS due to LVO (n = 33). Patients with AIS due to LVO were further divided into two subgroups, AIS with good collaterals (n = 17) and poor collaterals (n = 16)

An overview of the study design is shown in Fig. 1. Briefly, this study is a single-center, retrospective, observational case–control trial based on a prospectively collected stroke data base. Consecutive patients who presented at the Department of Neurology of Tongji Hospital were recruited from July 2016 to April 2018. The clinical diagnosis of acute ischemic stroke was confirmed by cranial magnetic resonance (MR) examinations with diffusion weighted imaging (DWI), and intracranial large vessel status was evaluated by digital subtraction angiography (DSA) within 7 days in every patient. Study-specific inclusion criteria were the presence of intracranial carotid artery terminus or first middle cerebral artery segment (M1) occlusion as confirmed on DSA, and sufficient display of the middle cerebral artery region. Study-specific exclusion criteria were the administration of thrombolysis and thrombectomy, and baseline modified Rankin Scale (mRS) > 1 before stroke onset. Patients with baseline mRS of 0–1 who missed therapeutic time window or refused acute reperfusion therapy were enrolled to rule out the influence from baseline clinical differences and acute reperfusion therapy. Collateral status was not an entry criterion for the trial and was assessed using the American Society of Interventional and Therapeutic Neuroradiology/Society of Interventional Radiology (ASTIN/SIR) grading system by blinded observers. Healthy volunteers with no prior history of stroke were matched with patients with AIS due to LVO for age and sex, and were recruited as a healthy control group, to control for the confounding effects of additional risk factors. Cranial MR with DWI and angiography were performed in healthy volunteers to verify that no silent brain infarcts or artery stenosis were present. Patients with AIS in the middle cerebral artery region but no obstructive large vessels were matched with patients with AIS due to LVO for age and sex, and enrolled as AIS due to non-LVO group.

In the discovery phase, there were 20 volunteers in the healthy control group and 40 patients with stroke due to LVO in the AIS group. 10 individual samples of equal volume from each group were pooled together for the proteomics analysis. In the validation phase, another independent population that included 33 healthy controls, 33 patients with AIS due to non-LVO, and 33 patients with AIS due to LVO were enrolled for Western blots analysis. The patients with AIS due to LVO were further divided into two groups based on their ASTIN/SIR grades: AIS with good collaterals (SGC, ASTIN/SIR 3–4), and AIS with poor collaterals (SPC, ASTIN/SIR 0–2). For prognostic prediction, patients with AIS due to LVO were divided into those with good prognosis (mRS 0–2) and those with poor prognosis (mRS 3–6) at a 3-month follow-up. Table 1 provides demographic details of the study participants.

**Table 1:** Baseline characteristics of patients with AIS and healthy control

Demographic and clinical characteristics of the study groups	Proteomics			Western Blots								
	Healthy controls (n = 20)	Patients with AIS (n = 40)	P value	Healthy controls (n = 33)	Patients with AIS (n = 66)	P value	Patients with AIS due to non-LVO (n = 33)	Patients with AIS due to LVO (n = 33)	P value	AIS with good collaterals (n = 17)	AIS with poor collaterals (n = 16)	P value
Characteristics												
Age, median (IQR), years	52 (47.5–59)	56 (47–63)	0.602	55 (45–62)	55 (48–63)	0.711	59 (51–65)	54 (46–59)	0.188	56 (53–59)	50.5 (43.5–59.75)	0.250
Female, n (%)	10 (50.0)	15 (37.5)	0.280	15 (45.5)	21 (31.8)	0.187	11 (33.3)	10 (30.3)	0.795	4 (23.5)	6 (37.5)	0.399
Systolic blood pressure, mmHg	129.0 (17.4)	141.5 (17.6)	0.015	129.9 (16.3)	145.9 (22.2)	0.000	149.1 (23.8)	142.7 (20.0)	0.247	146.2 (21.2)	139.0 (18.0)	0.319
Diastolic blood pressure, mmHg	77.5 (10.8)	82.2 (10.1)	0.115	79.6 (10.2)	85.5 (10.8)	0.012	86.8 (11.3)	84.2 (10.1)	0.339	86.6 (8.6)	81.7 (10.8)	0.172
Medical history, n (%)												
Hypertension	8 (40.0)	27 (67.5)	0.065	11 (33.3)	41 (62.1)	0.007	19 (57.6)	22 (66.7)	0.454	12 (70.6)	10 (62.5)	0.635
Diabetes mellitus	2 (10.0)	10 (25.0)	0.203	2 (6.1)	16 (24.2)	0.027	6 (18.2)	10 (30.3)	0.257	6 (35.3)	3 (18.8)	0.301
Coronary heart disease	3 (15.0)	4 (10.0)	0.529	5 (15.2)	5 (7.6)	0.243	2 (6.1)	3 (9.1)	0.458	3 (17.6)	0 (0.0)	0.082
Hypercholesterolemia	3 (15.0)	5 (12.5)	0.735	3 (9.1)	6 (9.1)	1.000	3 (9.1)	3 (9.1)	1.000	2 (11.8)	1 (6.3)	0.596
Current or previous smoking	7 (35.0)	18 (45.0)	0.561	9 (27.3)	32 (48.5)	0.044	16 (48.5)	16 (48.5)	1.000	10 (58.8)	6 (37.5)	0.233
Heavy alcohol use	2 (10.0)	10 (25.0)	0.203	6 (18.2)	22 (33.3)	0.117	11 (33.3)	11 (33.3)	1.000	7 (41.2)	4 (25.0)	0.340
Blood test, mean (SD)												
HCY	11.0 (4.3)	16.1 (8.9)	0.023	11.3 (3.7)	14.7 (6.6)	0.008	13.8 (5.2)	15.6 (7.6)	0.264	14.3 (7.2)	17.0 (7.8)	0.326
Cholesterol	3.9 (0.7)	3.6 (0.9)	0.364	3.9 (0.8)	3.9 (1.0)	0.980	4.1 (1.0)	3.6 (1.0)	0.075	3.7 (1.0)	3.6 (0.9)	0.795
Triglyceride	1.6 (0.9)	1.4 (0.7)	0.360	1.5 (0.8)	1.5 (1.0)	0.977	1.3 (0.6)	1.7 (1.3)	0.128	1.5 (0.8)	2.0 (1.6)	0.284
LDL	2.3 (0.6)	2.1 (0.8)	0.541	2.3 (0.7)	2.3 (0.9)	0.899	2.6 (0.9)	2.1 (0.7)	0.013	2.1 (0.7)	2.0 (0.7)	0.674
CRP	7.1 (17.3)	2.1 (0.8)	0.345	5.3 (13.8)	5.1 (8.2)	0.939	5.2 (7.0)	5.0 (9.3)	0.895	2.3 (4.2)	7.8 (12.1)	0.094

Data are shown as mean (SD), median (IQR) for continuous variables, and as percentages for categorical variables

*AIS* acute ischemic stroke, *IQR* interquartile range, *HCY* homocysteine, *LDL* low-density lipoprotein, *CRP* C-reactive protein

### Sample collection and preparation

Blood samples were drawn from subjects within 7 days after stroke onset and the healthy controls, and collected in EDTA Vacutainer tubes (BD, Oxford, UK), immediately put on ice, centrifuged at 1280×*g* for 10 min at 4 °C to separate plasma within 1 h, and stored at − 80 °C until required.

### iTRAQ-based quantitative proteomics analysis and Western blots validation

A detailed description of experimental procedures was provided in Additional file 1.

### Statistical analyses

Statistical analyses were performed using SPSS 19.0 for Windows. The continuous variables were described by mean ± SD or by medians and interquartile ranges (IQR), while categorical variables were described by percentages. Mann–Whitney U test, one-way analysis of variance (ANOVA) with Dunnett’s post hoc test were used to compare differences between groups. Adjusted logistic regression models were used to calculate the odds ratio (OR) and 95% confidence intervals (CI). Covariates included in the adjusted logistic regression models were determined by univariate logistic regression of every factor shown in Table 1. Logistic analysis and receiver operating characteristic (ROC) curves were employed to assess the diagnostic and prognostic accuracy of each biomarker. A combined ROC analysis was performed for panel diagnosis. All tests were considered statistically significant at P < 0.05.

## Results

### Clinical characteristics of patients

Baseline characteristic details of the patients and controls participating in the study are summarized in Table 1. For the discovery phase study, 40 patients with AIS due to LVO attending our hospital between July 2016 and April 2017 and 20 healthy control subjects were enrolled (Fig. 1). Compared with healthy controls, patients with AIS due to LVO had higher systolic blood pressure and homocysteinemia (HCY) levels. There were no significant differences in age, sex, medical history, or laboratory data between the two groups (Table 1).

For the validation phase study, 33 healthy controls, 33 patients with AIS due to non-LVO and 33 patients with AIS due to LVO were prospectively enrolled from April 2017 to April 2018 (Fig. 1). Higher systolic and diastolic blood pressure, higher HCY levels, higher hypertension and diabetes mellitus rates, as well as more patients with smoking history, were found in the AIS group than in the control group. Patients with AIS were divided into two subgroups: 33 patients with AIS due to LVO and 33 patients with AIS due to non-LVO. Lower low-density lipoprotein level was found in the LVO group. Patients with AIS due to LVO were further divided into two subgroups based on their ASTIN/SIR grades: 17 patients with good collaterals (SGC) and 16 patients with poor collaterals (SPC). There were no significant differences in demographic characteristics or laboratory data between the SGC and SPC groups (Table 1).

### Quantitative proteomic analysis reveals a specific proteomic profile in AIS due to LVO

We first compared plasma protein profiles from 40 consecutive patients with AIS due to LVO and 20 healthy controls. Pooled plasma samples were analyzed in replicates using 6-plex iTRAQ with LC–MS/MS. Patients with AIS due to LVO had a specific proteomic signature. The molecular features that were detected are depicted in the heatmap (Additional file 1: Figures S1 and S2). Seven differentially expressed proteins, thrombospondin 1 (THBS1), secreted phosphoprotein 2 (SPP2), lymphatic vessel endothelial hyaluronic acid receptor 1 (LYVE1), cadherin 1 (CDH1), insulin-like growth factor 2 (IGF2), pro-platelet basic protein (PPBP), and apolipoprotein C 4-2 (APOC4-APOC2), were finally acquired with a 1.2/0.83-fold or greater difference in abundance in AIS samples compared to healthy controls (Additional file 1: Table S1). Of these seven proteins, THBS1, LYVE1, IGF2, and PPBP were more abundant in AIS due to LVO samples, while SPP2, CDH1, and APOC4-APOC2 were downregulated compared to control samples.

### Validation of potential biomarkers by Western blots analysis

**Fig. 2:**
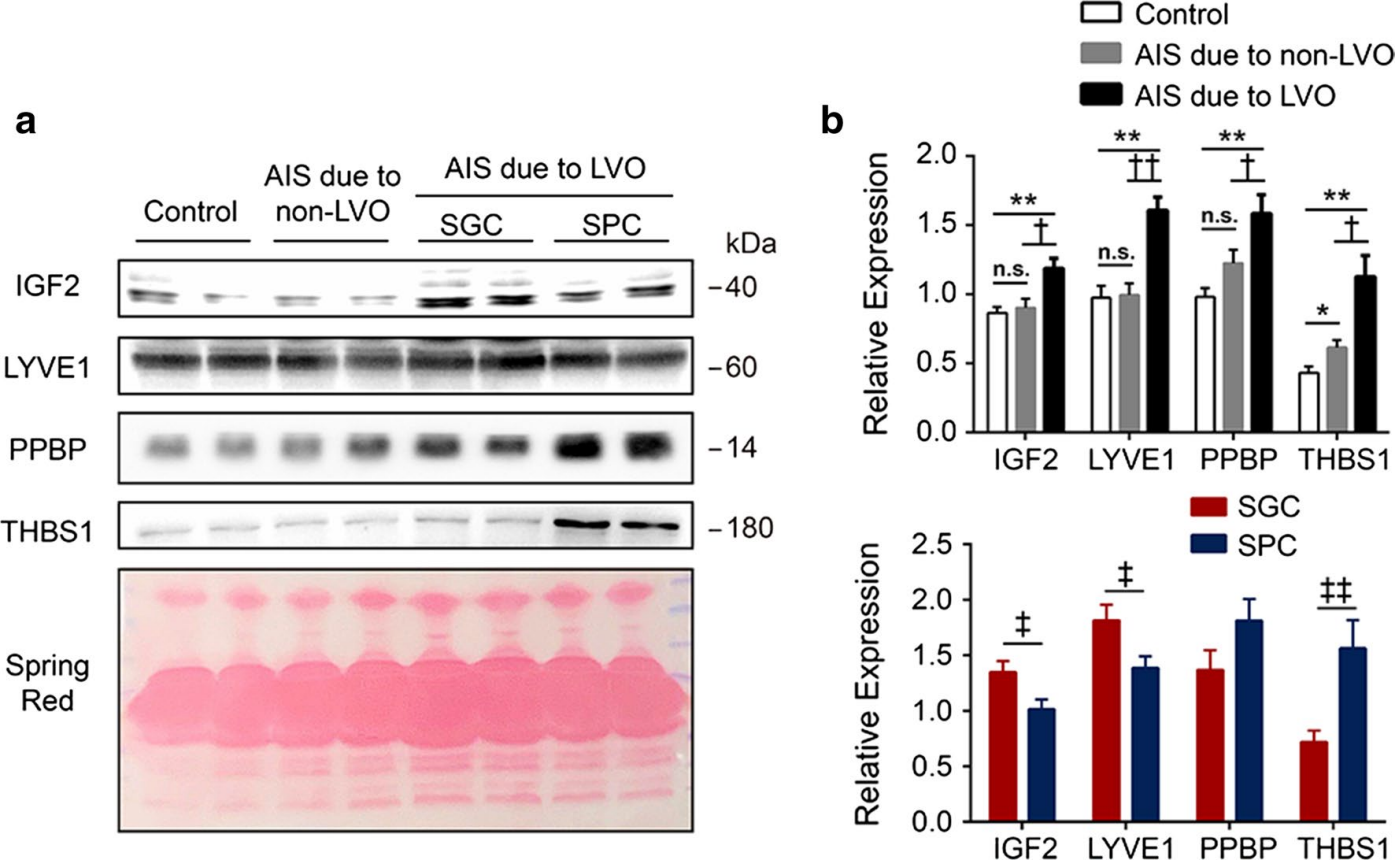
Validation phase of potential biomarkers by Western blots analysis. **a** Western blots representation of each potential biomarker. **b** Quantitative analysis was performed. *P < 0.05 **P  < 0.01 versus Control, ^†^P < 0.05 ^††^P < 0.01 versus AIS due to non-LVO, n = 33 for Control, n = 33 for AIS due to non-LVO group and n = 33 for AIS due to LVO group. ^‡^P < 0.05 ^‡‡^P < 0.01 versus SGC, n = 17 for SGC, n = 16 for SPC. *Control* healthy controls. *AIS* acute ischemic stroke patients, *SGC* stroke with good collaterals, *SPC* stroke with poor collaterals

Interestingly, among these differentially expressed proteins identified in plasma samples of AIS due to LVO, all the upregulated proteins, IGF2, LYVE1, PPBP, and THBS1, are known to be involved in regulating blood hemostasis and endothelial function [6–11]. We then verified the differential abundance of four selected proteins (IGF2, LYVE1, PPBP, and THBS1) using Western blots analysis. Consistent with the tendency found in proteomics, Western blots results showed similar alterations in an independent population, as the levels of those four identified proteins were significantly higher in the AIS due to LVO group compared to the healthy control group (Fig. 2, Tables 2 and 3). To describe the specificity for AIS due to LVO, we then detected the levels of the four identified proteins in patients with AIS due to non-LVO. Interestingly, expression of IGF2, LYVE1 and PPBP showed no significant difference between AIS due to non-LVO and healthy control, except that THBS1 was higher in AIS due to non-LVO group. Further, comparisons between the non-LVO and LVO groups presented higher expression of IGF2, LYVE1, PPBP and THBS1 in the LVO group (Fig. 2, Table 2). Altered levels of these proteins might indicate endothelial dysfunction and clinical implications of large vessel occlusion.

**Table 2:** Plasma levels of each biomarker in different groups

Proteins	Healthy controls (n = 33)	Patients with AIS due to non-LVO (n = 33)	Patients with AIS due to LVO (n = 33)	P value	P^a^ value	P^b^ value	P^c^ value
IGF2	0.86 ± 0.24	0.90 ± 0.50	1.19 ± 0.42	0.001	0.948	0.001	0.017
LYVE1	0.97 ± 0.50	0.99 ± 0.49	1.61 ± 0.56	< 0.001	0.998	< 0.001	< 0.001
PPBP	0.98 ± 0.37	1.23 ± 0.55	1.58 ± 0.78	< 0.001	0.081	0.001	0.045
THBS1	0.43 ± 0.26	0.62 ± 0.29	1.13 ± 0.88	< 0.001	0.030	< 0.001	0.010

Data are shown as mean ± SD. One-way analysis of variance (ANOVA) with Dunnett’s post hoc test

*AIS* acute ischemic stroke, *LVO* large vessel occlusion

^a^Patients with AIS due to non-LVO versus healthy controls

^b^Patients with AIS due to LVO versus healthy controls

^c^Patients with AIS due to LVO versus patients with AIS due to non-LVO

**Table 3:** Correlation analysis of each biomarker with diagnosis

Proteins	OR^a^ (95% CI^a^)	P^a^ value	OR^b^ (95% CI^b^)	P^b^ value	AUC (95% CI)	P^c^ value
IGF2	17.365 (2.862–105.358)	0.002	47.564 (5.133–440.757)	0.001	0.731 (0.609–0.853)	0.001
LYVE1	10.320 (2.986–35.669)	< 0.001	7.707 (2.215–26.813)	0.001	0.813 (0.707–0.919)	< 0.001
PPBP	7.682 (2.201–26.813)	0.001	8.163 (1.903–35.014)	0.005	0.754 (0.638–0.870)	< 0.001
THBS1	13.282 (2.630–67.079)	0.002	14.132 (2.401–83.185)	0.003	0.769 (0.652–0.885)	< 0.001

*AUC* area under the receiver operating characteristic curve, *OR* odds ratio, *CI* confidence interval, *IGF2* insulin like growth factor 2, *LYVE1* lymphatic vessel endothelial hyaluronan receptor 1, *PPBP* pro-platelet basic protein, *THBS1* thrombospondin 1

^a^Logistic regression analysis, OR and 95% CI without adjusting

^b^Logistic regression analysis, OR and 95% CI with adjusting for age, gender, blood pressure, medical history and blood test

^c^Receiver operating characteristic curve analysis

Patients with AIS due to LVO were further divided into SGC and SPC subgroups, according to the ASTIN/SIR grades assigned to their collaterals. Comparisons between the SGC and SPC group indicated higher expression of LYVE1 and IGF2 in the SGC group and higher level of THBS1 in the SPC group, but no significant difference in PPBP (Fig. 2, Table 4).

**Table 4:** Correlation analysis of each biomarker with prognosis in AIS with different collaterals

Proteins	AIS with good collaterals (n = 17)	AIS with poor collaterals (n = 16)	P value	OR^a^ (95% CI^a^)	P^a^ value	OR^b^ (95% CI^b^)	P^b^ value
IGF2	1.35 ± 0.41	1.02 ± 0.35	0.022	0.115 (0.016–0.841)	0.033	0.115 (0.015–0.866)	0.036
LYVE1	1.82 ± 0.58	1.39 ± 0.42	0.026	0.183 (0.036–0.918)	0.039	0.028 (0.002–0.334)	0.005
PPBP	1.37 ± 0.73	1.81 ± 0.77	0.111	1.939 (0.707–5.321)	0.198	2.806 (0.933–8.439)	0.066
THBS1	0.72 ± 0.42	1.56 ± 1.02	0.005	4.257 (1.273–14.228)	0.019	3.294 (1.158–9.372)	0.025

Data are shown as mean ± SD

*OR* odds ratio, *CI* confidence interval, *IGF2* insulin like growth factor 2, *LYVE1* lymphatic vessel endothelial hyaluronan receptor 1, *PPBP* pro-platelet basic protein, *THBS1* thrombospondin 1

^a^Logistic regression analysis of each biomarker with different collaterals, OR and 95% CI with adjusting for age, gender, blood pressure, medical history and blood test

^b^Logistic regression analysis each biomarker with prognosis, OR and 95% CI with adjusting for age, gender, blood pressure, medical history and blood test

### Predictive potential of biomarkers

In order to define the capacity of these potential biomarkers for diagnosis, logistic regression and receiver operating characteristic (ROC) curves were employed. Logistic analysis results supported the role of plasma levels of IGF2, LYVE1, PPBP, and THBS1 as potential biomarkers that indicate a significantly increased risk of AIS due to LVO (Table 3).

**Fig. 3:**
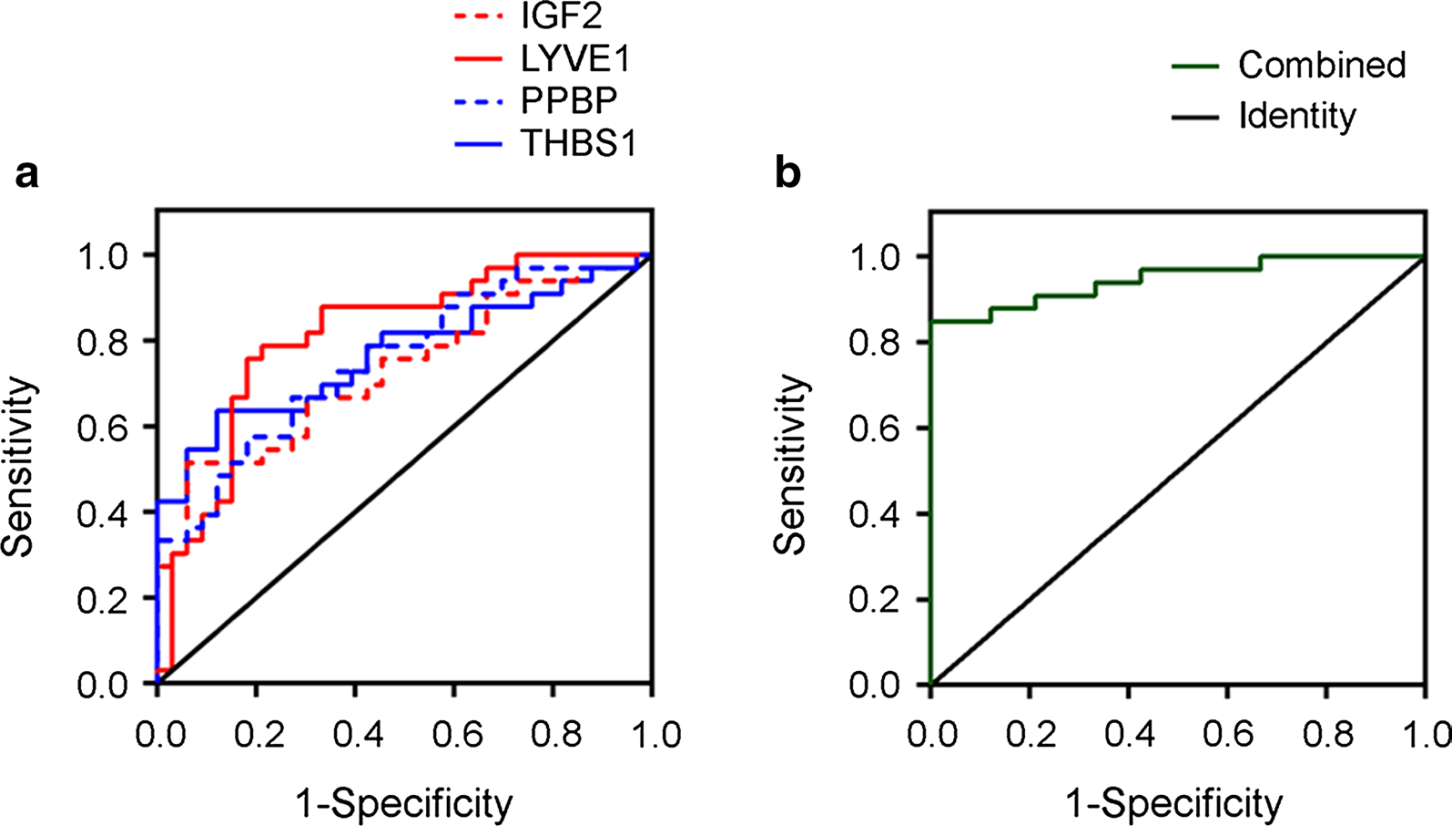
Diagnostic efficiency of the potential biomarkers. **a** Receiver operating characteristic curve of biomarkers in diagnosis of AIS due to LVO. IGF2, LYVE1, PPBP and THBS1 could be efficient in diagnosis, and **b** 4-protein panel combined has better performance

We then assessed the diagnostic efficiency of the potential biomarkers using ROC analysis. The areas under the ROC curve (AUC) were 0.731 (0.609–0.853) for IGF2, 0.813 (0.707–0.919) for LYVE1, 0.754 (0.638–0.870) for PPBP, and 0.769 (0.652–0.885) for THBS1, respectively (Fig. 3a, Table 3). Furthermore, the diagnostic potential of the 4-protein panel was also evaluated. The AUC value of the panel reached 0.947 for distinguishing patients with AIS due to LVO from healthy individuals, which was interpreted as excellent classification performance (Fig. 3b).

We further investigated the association between plasma levels of these identified potential biomarkers and the prognosis of AIS due to LVO. Patients were divided into those with good prognosis (modified Rankin scale, mRS 0–2) and those with poor prognosis (mRS 3–6) at a 3-month follow-up. Logistic analysis results showed that higher IGF2 and LYVE1 levels predicted favorable outcomes, while higher THBS1 levels were related to a poor prognosis. The results implicated that the three collateral-related proteins (IGF2, LYVE1, and THBS1) were prognostic for patients with AIS due to LVO, while PPBP showed no potential for outcome prediction (Table 4).

## Discussion

The aim of this study was to analyze plasma proteomic profiles of patients with AIS due to LVO, and to identify potential biomarkers that are associated with the status of collateral circulation. This study yielded two main findings. First, four upregulated proteins, IGF2, LYVE1, PPBP, and THBS1, were identified in patients with AIS due to LVO, compared to healthy controls and patients with AIS due to non-LVO. Second, IGF2, LYVE1, and THBS1 were closely associated with collateral status, and were shown to be independent predictors of favorable outcome at a 3-month follow-up.

Insulin-like growth factor 2 (IGF2) was found to be required for angiogenesis in growing tumors via the induction of hypoxia-related vascular endothelial growth factor [7]. Although no much evidence in the association between IGF2 and the risk of ischemic stroke yet, IGF2 might contribute to vascular remodeling and neovascularization by enhancing the recruitment and incorporation of endothelial progenitor cells into areas of ischemic injury [7].

Lymphatic vessel endothelial hyaluronic acid receptor 1 (LYVE1), a cell surface receptor on lymphatic endothelial cells, is involved in capillary sprouting through lymphatic/blood endothelial cell interactions [8]. LYVE1 levels have been reported significantly increased in patients with malignant middle cerebral artery infarction, and indicated as a potential marker of early prediction [12].

Pro-platelet basic protein (PPBP) can be released in large amounts from activated platelets [13], and is a key participant in both homeostasis and inflammation during ischemia [9, 10]. PPBP has been identified as an inflammatory biomarker increased in central retinal vein ischemic occlusion [9], and as a potential risk factor of coronary heart disease in patients with hyperlipidaemia [10].

Thrombospondin 1 (THBS1) was first isolated from human blood platelets as a thrombin-sensitive protein [14]. THBS1 is a potent regulator of angiogenesis that functions to concurrently inhibit endothelial cell migration and the release of vascular endothelial growth factor from the extracellular matrix [11]. Cerebral THBS1 expression is upregulated in experimental focal cerebral ischemia [15] and the circulating THBS1 were elevated in patients with ischemic stroke [14].

The proteomic signature of patients with AIS due to LVO in our study is characterized by the increase in these four proteins, when compared to both healthy controls and AIS due to non-LVO group. Large vessel occlusion may explain the change, as the four proteins are involved in regulating blood hemostasis and endothelial function. The combination of the four biomarkers could robustly identify (with an accuracy > 90%) these patients, offering excellent predictive values for distinguishing acute LVO strokes. Blood is easy to obtain, making this biomarker panel a useful predictor that could be screened for in large-scale clinical trials and for future clinical use.

Collateral circulation refers to the network of blood vessels that contribute towards maintaining blood flow in the absence of the primary conduit [16]. The degree of such collateral blood supply varies amongst individuals and can influence outcomes, especially in patients with AIS due to LVO [16]. Patients with good collateral flow demonstrated less hypoperfused tissue, less infarct growth within the penumbra zone, and smaller infarct volumes with better outcomes than those with poor collaterals [17]. Our study found that elevated IGF2 and LYVE1 levels and lower THBS1 levels were associated with better collateral circulation in patients with AIS due to LVO. No significant difference was observed in PPBP. Interestingly, the three collateral-related proteins (IGF2, LYVE1, and THBS1) were prognostic, as patients with relatively higher IGF2 and LYVE1 levels and lower THBS1 levels were more likely to have good outcomes, while PPBP showed no potential as a predictor of outcomes. IGF2 and LYVE1 are key determinants in vascular remodeling and neovascularization [6–8], and THBS1 has been shown to inhibit angiogenesis [11]. Despite the relationship between endothelium function and collateral circulation is complex, our findings suggest that IGF2, LYVE1, and THBS1 could be associated with cerebral collateral circulation and thus clinical outcomes after AIS due to LVO, making them potential predictors.

## Limitations

The present study was subject to several limitations. The small sample size and the retrospective nature of the study indicate that biases are inevitable. Moreover, all patients were ethnically Chinese; our results may not apply to other populations. Further research on the dynamic changes of the four biomarkers from hyper-acute phase to chronic phase of ischemic stroke is needed to elucidate details concerning the diagnostic and prognostic values of these biomarkers in stroke. The development of clinical trials in large scale population and long term assessment is also warranted.

## Conclusions

To our knowledge, this is the first study of a blood-based biomarker panel with high accuracy for detecting AIS due to LVO. Although actual mechanisms of collateral blood flow regulation remain elusive, the collateral-related proteins found here showed potential as predictors of clinical outcomes of patients with AIS due to LVO at a 3-month follow-up. Both the diagnostic biomarker panel and the prognostic predictors require external validation in a more diverse demographic group with longer follow-up assessment before further development for clinical use.

## Additional file

**Additional file 1.** Proteomic profiling of plasma biomarkers in acute ischemic stroke due to large vessel occlusion.

## Data Availability

The datasets used and analysed during the current study are available from the corresponding author on reasonable request.

## Abbreviations

AIS: acute ischemic stroke
LVO: large vessel occlusions
SGC: AIS with good collaterals
SPC: AIS with poor collaterals
DSA: digital subtraction angiography
ROC: receiver operating characteristic
AUC: areas under the ROC curve
OR: odds ratio
CI: confidence interval
IGF2: insulin like growth factor 2
LYVE1: lymphatic vessel endothelial hyaluronan receptor 1
PPBP: pro-platelet basic protein
THBS1: thrombospondin 1
