# Supplementary material for: Proteomic profiling of plasma biomarkers in acute ischemic stroke due to large vessel occlusion
Source: J Transl Med. 2019 Jul 1;17:214. doi: 10.1186/s12967-019-1962-8 (PMC6604304; doi:10.1186/s12967-019-1962-8)
Supplement: Supplementary file 1 — Additional file 1. Proteomic profiling of plasma biomarkers in acute ischemic stroke due to large vessel occlusion. [file 12967_2019_1962_MOESM1_ESM.pdf]

Additional Files

Additional Methods

Sample collection and preparation

Blood samples were drawn from subjects within 7 days after stroke onset and the healthy controls, and collected in EDTA Vacutainer tubes (BD, Oxford, UK), immediately put on ice, centrifuged at 1280 g for 10 minutes at 4 °C to separate plasma within 1 hour, and stored at -80 °C until required.

For the proteomics analysis, 10 individual samples of equal volume from each group were pooled together, and total proteins were extracted using the cold acetone method. Briefly, samples were ground to powder in liquid nitrogen, then dissolved in 2 mL lysis buffer (8 M urea, 2% SDS, 1 x Protease Inhibitor Cocktail (Roche Ltd., Basel, Switzerland), followed by sonication on ice for 30 minutes and centrifugation at 4180 g for 30 minutes at 4 °C. The supernatant was precipitated with ice-cold acetone at -20 °C overnight. The precipitations were cleaned with acetone three times and re-dissolved in 8 M urea by sonication on ice. Protein quality was examined with sodium dodecylsulfate-polyacrylamide gel electrophoresis (SDS-PAGE). For Western blots validation, each plasma sample was diluted 1:5 in PBS.

iTRAQ-based quantitative proteomics analysis

The proteomics analysis was performed by Guangzhou Gene Denovo Co., Ltd. (Guangzhou, China) and included protein digestion, iTRAQ labelling, strong cation exchange (SCX) fractionation, LC-MS/MS analysis, protein identification, and protein quantitation.

Protein digestion and iTRAQ labelling

BCA protein assays were used to determine the protein concentration of the supernatant. Per condition, 100 μg protein were transferred into a new tube and adjusted to a final volume of 100 μL with 8 M urea. Then, 11 μL of 1 M DTT (DL-Dithiothreitol) was added and samples were incubated at 37 °C for 1 hour. Finally, 120 μL of the 55 mM iodoacetamide was added to the sample and incubated for 20 minutes, protected from light, at room temperature.

For each sample, proteins were precipitated with ice-cold acetone, then re-dissolved in 100 μL TEAB. Proteins were then tryptic digested with sequence-grade modified trypsin (Promega, Madison, WI, USA) at 37 °C overnight. The resultant peptide mixture was labeled with iTRAQ tags 113 to 118, as shown in **Figure 1**. The labeled samples were combined and dried under vacuum.

Strong cation exchange (SCX) fractionation and liquid chromatography-tandem mass spectrometry (LC-MS/MS) analysis

The combined labeled samples were subjected to the SCX fractionation column connected with a high-performance liquid chromatography (HPLC) system. The peptide mixture was re-dissolved in buffer A (buffer A: 20 mM ammonium formate in water, pH 10.0, adjusted with ammonium hydroxide), and then fractionated by high-pH separation using an Ultimate 3000 system (Thermo Fisher scientific, MA, USA) connected to a reverse phase column (XBridge

C18 column, 4.6 mm x 250 mm, 5 µm; Waters Corporation, MA, USA). High-pH separation was performed using a linear gradient starting from 5% B to 45% B in 40 minutes (B: 20 mM ammonium formate in 80% ACN, pH 10.0, adjusted with ammonium hydroxide). The column was re-equilibrated at initial conditions for 15 minutes. The column flow rate was maintained at 1 mL/minute and column temperature was maintained at 30 °C. Twelve fractions were collected; each fraction was dried in a vacuum concentrator for the next step.

Peptide fractions were resuspended with 30 µL solvent C respectively (C: water with 0.1% formic acid; D: ACN with 0.1% formic acid), separated by nanoLC and analyzed by on-line electrospray tandem mass spectrometry. The experiments were performed on an Easy-nLC 1000 system (Thermo Fisher Scientific) connected to an Orbitrap Fusion Tribrid mass spectrometer (Thermo Fisher Scientific) equipped with an online nano-electrospray ion source. Of the peptide sample, 10 µL was loaded onto the trap column (Thermo Scientific Acclaim PepMap C18, 100 µm x 2 cm), with a flow of 10 µL/minute for 3 minutes, and subsequently separated on the analytical column (Acclaim PepMap C18, 75 µm x 15 cm) with a linear gradient, from 3% D to 32% D in 120 minutes. The column was re-equilibrated at initial conditions for 10 minutes. The column flow rate was maintained at 300 nL/minute. An electrospray voltage of 2 kV versus the inlet of the mass spectrometer was used.

The fusion mass spectrometer was operated in the data-dependent mode and switched automatically between MS and MS/MS acquisition. Survey full-scan MS spectra (m/z 350–1550) were acquired with a mass resolution of 120 K, followed by sequential high-energy collisional dissociation (HCD) MS/MS scans with a resolution of 30 K. The isolation window was set as 1.6 Da. The AGC target was set to 400,000. MS/MS fixed first mass was set to 110. In all cases, one microscan was recorded using a dynamic exclusion of 45 seconds.

Bioinformatic analysis

Database search

The mass spectrometry data were transformed into MGF files with Proteome Discovery 1.2 (Thermo, Pittsburgh, PA, USA) and analyzed using the Mascot search engine (Matrix Science, London, UK; version 2.3.2). The Mascot database was set up for protein identification using “human reference transcriptome” or “human database” in NCBInr/SwissProt/Uniprot/IPI and searched using a fragment ion mass tolerance of 0.050 Da and a parent ion tolerance of 10.0 PPM.

Protein identification and quantification

The Mascot search results were averaged using medians and then quantified. Proteins with fold differences of >1.2 or <0.83 between groups and an unadjusted significance level $p<0.05$ were considered differentially expressed.

Protein functional annotation and enrichment analysis

Proteins were annotated against the GO, KEGG, and COG/KOG databases, in order to reveal their functions. Significant GO functions and pathways were examined within differentially expressed proteins with a $p \leq 0.05$.

Western blots validation

The plasma levels of selected differentially regulated proteins were measured by Western blots analysis. Briefly, the concentration of plasma protein was measured using bovine serum albumin (BSA, Beyotime, China) as a standard. A portion (20 μg) of each sample extract was then resolved on 12% SDS-PAGE gel electrophoresis, blotted, and incubated overnight with the primary antibodies against IGF-2 (1:1000, ab177467, Abcam, UK), THBS-1 (1:200, SC-59887, Santa Cruz Biotechnology, USA), PPBP (1:500, A1925, ABclonal, USA), and LYVE-1 (1:2000, af2089, R&D Systems, USA). Then, membranes were incubated for 1 hour at room temperature with horseradish peroxidase-labeled anti-mouse, anti-goat, or anti-rabbit secondary antibody (1:5000, Boster, China). The membranes were visualized with enhanced chemiluminescence kits (Thermo Fisher Scientific), evaluated via a CCD camera system, and the intensity of blots was semi-quantified using ImageJ. The membranes were stained with Spring red as the internal control.

Additional Results

Categorization and functional annotation of differentially regulated proteins in AIS due to LVO

We then categorized the differentially regulated proteins on the basis of their gene ontology (GO), including biological processes, cellular components, and molecular functions (**Additional Fig. 2a**). Based on the level 2 GO terms of their corresponding transcripts, the proteins identified were found to be involved in several important biological processes such as biological adhesion (7.45%), localization (10.64%), reproduction (3.72%), signaling (7.98%), developmental processes (8.51%), immune system processes (7.45%), multicellular organismal processes (3.72%), stimulus responses (10.10%), biological regulation (7.98%), metabolic processes (7.98%), cellular component organization or biogenesis (6.91%), single-organism processes (8.51%), cellular processes (7.98%), and locomotion (1.06%). The proteins were mainly expressed in extracellular region parts (18.58%), membranes (12.39%), extracellular region parts (11.50%), macromolecular complexes (8.85%), cell parts (16.81%), cells (16.81%), and organelles (15.04%). Their molecular functions included molecular function regulation (45.45%), transporter activity (4.55%), signal transducer activity (4.55%), and binding (45.45%).

Further, we analyzed the pathways involved using the Kyoto Encyclopedia of Genes and Genomes (KEGG) classification. In total, 22 differential KEGG pathways were identified in the enrichment analysis (**Additional Table 2**). The "Top 10" pathways mainly focused on cancers, adherens junctions, the Hippo signaling pathway, pathogenic Escherichia coli infections, bacterial invasions of epithelial cells, and the Rap1 signaling pathway (**Additional Fig. 2b**). We then analyzed the protein-protein interaction (PPI) network of the seven differentially expressed proteins using a string database. PPBP, THBS1, and IGF2 were found to be at the central position of the PPI network and to interact with each other (**Additional Fig. 2c**).

Additional Figures and Figure Legends

Additional Fig. 1

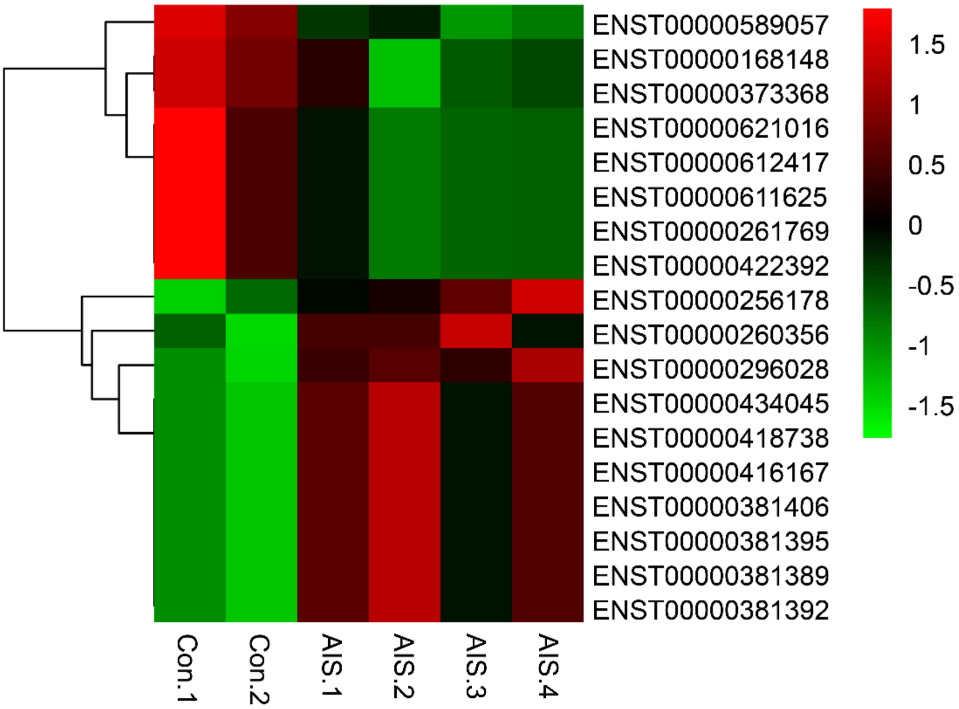

Additional Fig. 1

Heatmap representation of hierarchical clustering of molecular features found in each pooled plasma sample.

Each line of this graphic represents molecular profile of pooled sample of 10 individual plasma, colored by its abundance intensity and baselining to median/mean across the samples. The scale from -1.5 green (low abundance) to 1.5 red (high abundance) represents this normalized abundance in arbitrary units. Con, Healthy Control. AIS, Acute Ischemic Stroke.

Additional Fig. 2

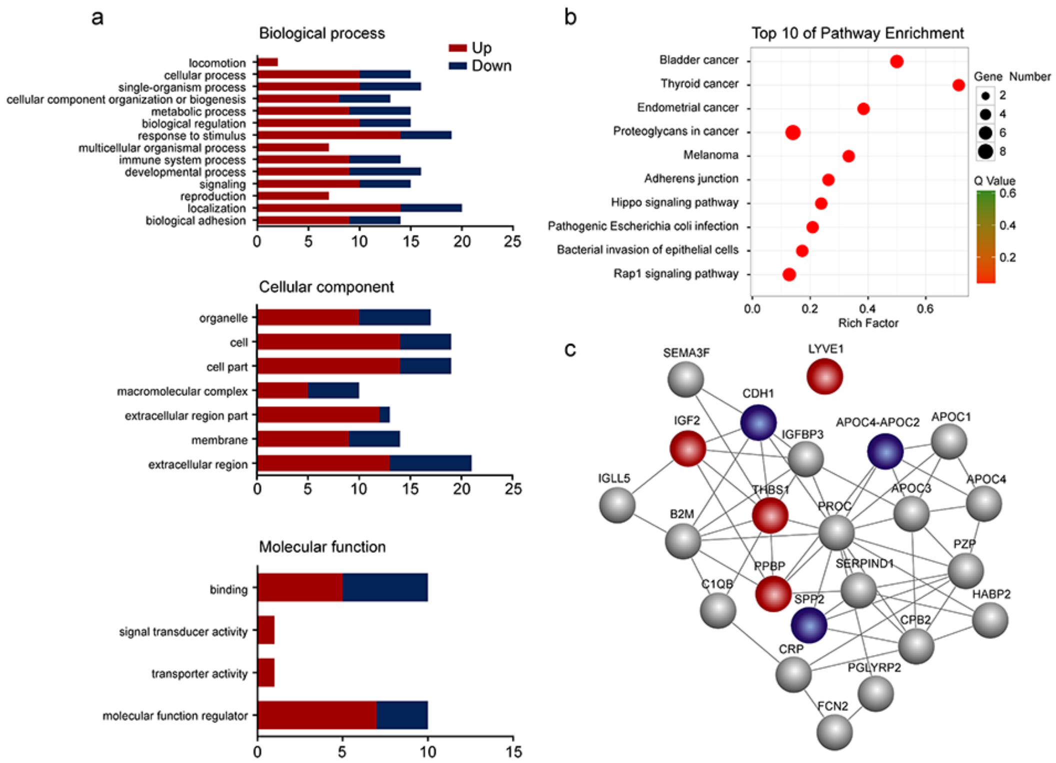

Additional Fig. 2

Categorization and functional annotation of differentially regulated proteins in AIS due to LVO.

a. Categorization and functional annotation of differentially regulated transcripts based on gene ontology, including biological process, cellular component and molecular function. **b.** Top 10 differential KEGG pathways identified in the enrichment analysis. **c.** Protein-protein interaction network of the 7 differentially expressed proteins using string database.

Additional Table 1 Candidate Proteins Identified in AIS patients due to LVO

Symbol	Protein Name	Control	AIS due to LVO	Fold change	P value
APOC4-APOC2	APOC4-APOC2 readthrough	0.942	0.591	0.626857749	0.011255
CDH1	cadherin 1, type 1	0.935	0.756	0.80828877	0.016479
IGF2	insulin like growth factor 2	0.973	1.218	1.251284687	0.012312
LYVE1	lymphatic vessel endothelial hyaluronan receptor 1	1.106	1.609	1.455676165	0.027026
PPBP	pro-platelet basic protein	0.938	1.379	1.470933333	0.003772
SPP2	secreted phosphoprotein 2	0.961	0.747	0.777055151	0.046845
THBS1	thrombospondin 1	0.891	1.306	1.466872544	0.029978

Control, healthy control; AIS, Acute Ischemic Stroke; LVO, large vessel occlusion; APOC, Apolipoprotein C; CDH1, cadherin 1, type 1; IGF2, insulin like growth factor 2; LYVE1, lymphatic vessel endothelial hyaluronan receptor 1; PPBP, pro-platelet basic protein; SPP2, secreted phosphoprotein 2; THBS1, thrombospondin 1.

Additional Table 2 Differential KEGG pathways identified in the enrichment analysis

Symbol	Pathway	KEGG_A_class	KEGG_B_class
**APOC4-APOC2**	-	-	-
**CDH1**		Cellular Processes;<br>Human Diseases;<br>Environmental Information Processing	Cancers;<br>Cellular commiuniuty;<br>Infectious diseases;<br>Signal transduction;<br>Signaling molecules and interaction
	ko05200//Pathways in cancer;<br>ko04015//Rap1 signaling pathway;<br>ko04390//Hippo signaling pathway;<br>ko04514//Cell adhesion molecules (CAMs);<br>ko04520//Adherens junction;	Human Diseases;<br>Environmental Information Processing;<br>Cellular Processes	Signaling molecules and interaction;<br>Signal transduction;<br>Infectious diseases;<br>Cellular commiuniuty;<br>Cancers
	ko05100//Bacterial invasion of epithelial cells;<br>ko05218//Melanoma;<br>ko05213//Endometrial cancer;<br>ko05130//Pathogenic Escherichia coli infection;<br>ko05219//Bladder cancer;ko05216//Thyroid cancer	Environmental Information Processing;<br>Human Diseases;<br>Cellular Processes	Cellular commiuniuty;<br>Cancers;<br>Signaling molecules and interaction;<br>Infectious diseases;<br>Signal transduction
		Cellular Processes;<br>Environmental Information Processing;<br>Human Diseases	Signal transduction;<br>Infectious diseases;<br>Signaling molecules and interaction;<br>Cancers;<br>Cellular commiuniuty
		Cellular Processes;	Cancers;

		Human Diseases;<br>Environmental Information Processing	Cellular commiunity;<br>Signal transduction;<br>Infectious diseases;<br>Signaling molecules and interaction
**SPP2**	-	-	-
**PPBP**	ko04060//Cytokine-cytokine receptor interaction;<br>ko04062//Chemokine signaling pathway	Organismal Systems;<br>Environmental Information Processing	Immune system;<br>Signaling molecules and interaction
**IGF2**	ko05205//Proteoglycans in cancer	Human Diseases	Cancers
**LYVE1**	-	-	-
**THBS1**	ko04151//PI3K-Akt signaling pathway;<br>ko04015//Rap1 signaling pathway;<br>ko05205//Proteoglycans in cancer;<br>ko04510//Focal adhesion;<br>ko05206//MicroRNAs in cancer;<br>ko04145//Phagosome;<br>ko04115//p53 signaling pathway;<br>ko04350//TGF-beta signaling pathway;<br>ko04512//ECM-receptor interaction;<br>ko05219//Bladder cancer;<br>ko05144//Malaria	Cellular Processes;<br>Human Diseases;<br>Environmental Information Processing	Signaling molecules and interaction;<br>Transport and catabolism;<br>Signal transduction;<br>Infectious diseases;<br>Cellular commiunity;<br>Cancers;<br>Cell growth and death

KEGG, Kyoto Encyclopedia of Genes and Genomes; APOC, Apolipoprotein C; CDH1, cadherin 1, type 1; SPP2, secreted phosphoprotein 2; PPBP, pro-platelet basic protein; IGF2, insulin like growth factor 2; LYVE1, lymphatic vessel endothelial hyaluronan receptor 1; THBS1, thrombospondin 1
